# Sustainable chromatographic quantitation of multi-antihypertensive medications: application on diverse combinations containing hydrochlorothiazide along with LC–MS/MS profiling of potential impurities: greenness and whiteness evaluation

**DOI:** 10.1186/s13065-023-01015-z

**Published:** 2023-08-19

**Authors:** Hoda M. Marzouk, Sara El-Hanboushy, Reem H. Obaydo, Yasmin M. Fayez, Mohamed Abdelkawy, Hayam M. Lotfy

**Affiliations:** 1 Analytical Chemistry Department, Faculty of Pharmacy, Cairo University, Kasr El-Aini Street, Cairo, 11562 Egypt; 2 Pharmaceutical Chemistry Department, Faculty of Pharmacy, Future University in Egypt, Cairo, 12311 Egypt; 3 Analytical and Food Chemistry Department, Faculty of Pharmacy, Ebla Private University, 22743 Idlib, Syria

**Keywords:** Antihypertensive drugs, Impurity profiling, Diverse pharmaceutical combinations, HPLC–DAD, LC–MS/MS, Greenness and whiteness evaluation

## Abstract

Cardiovascular disorders are among the leading causes of death worldwide, especially hypertension, a silent killer syndrome requiring multiple drug therapy for appropriate management. Hydrochlorothiazide is an extensively utilized thiazide diuretic that combines with several antihypertensive drugs for effective treatment of hypertension. In this study, sustainable, innovative and accurate high performance liquid chromatographic methods with diode array and tandem mass detectors (HPLC–DAD and LC–MS/MS) were developed, optimized and validated for the concurrent determination of Hydrochlorothiazide (HCT) along with five antihypertensive drugs, namely; Valsartan (VAL), Amlodipine besylate (AML), Atenolol (ATN), Amiloride hydrochloride (AMI), and Candesartan cilextil (CAN) in their diverse pharmaceutical dosage forms and in the presence of Chlorothiazide (CT) and Salamide (DSA) as HCT officially identified impurities. The HPLC–DAD separation was achieved utilizing Inertsil ODS-3 C_18_ column (250 × 4.6 mm, 5 μm) attached with photodiode array detection at 225.0 nm. Gradient elution was performed utilizing a mixture of solvent A (20.0 mM potassium dihydrogen phosphate, pH 3.0 ± 0.2, adjusted with phosphoric acid) and solvent B (acetonitrile) at ambient temperature. Linearity ranges were 0.1–100.0 µg/mL for HCT, VAL, AML and CAN, 0.05 –100.0 µg/mL for both ATN and AMI and 0.05–8.0 µg/mL for both CT and DSA. Additionally, this work describes the use of liquid chromatography–electrospray–tandem mass spectrometry for the accurate detection and quantification of the impurities; CT and DSA in the negative mode utilizing triple quadrupole mass spectrometry. The linearity ranges for those impurities were 1.0–200.0 ng/mL and 5.0–200.0 ng/mL for CT and DSA, respectively. Developed methods’ validation was achieved in accordance with International Conference on Harmonization (ICH) guidelines. Upon applying liquid chromatographic techniques for the drug analysis, a green and sustainable assessment have to be handled due to the consumption of energy and many solvents. Through the use of the HEXAGON, Analytical Greenness (AGREE) and White Analytical Chemistry (WAC) tools, greenness and sustainability have been statistically assessed. The optimized HPLC–DAD and LC–MS/MS methods were fast, accurate, precise, and sensitive, and consequently could be applied for conventional analysis and quality control of the proposed drugs in their miscellaneous dosage forms for the purpose of reducing laboratory wastes, time of the analysis time, effort, and cost.

**Graphical Abstract:**

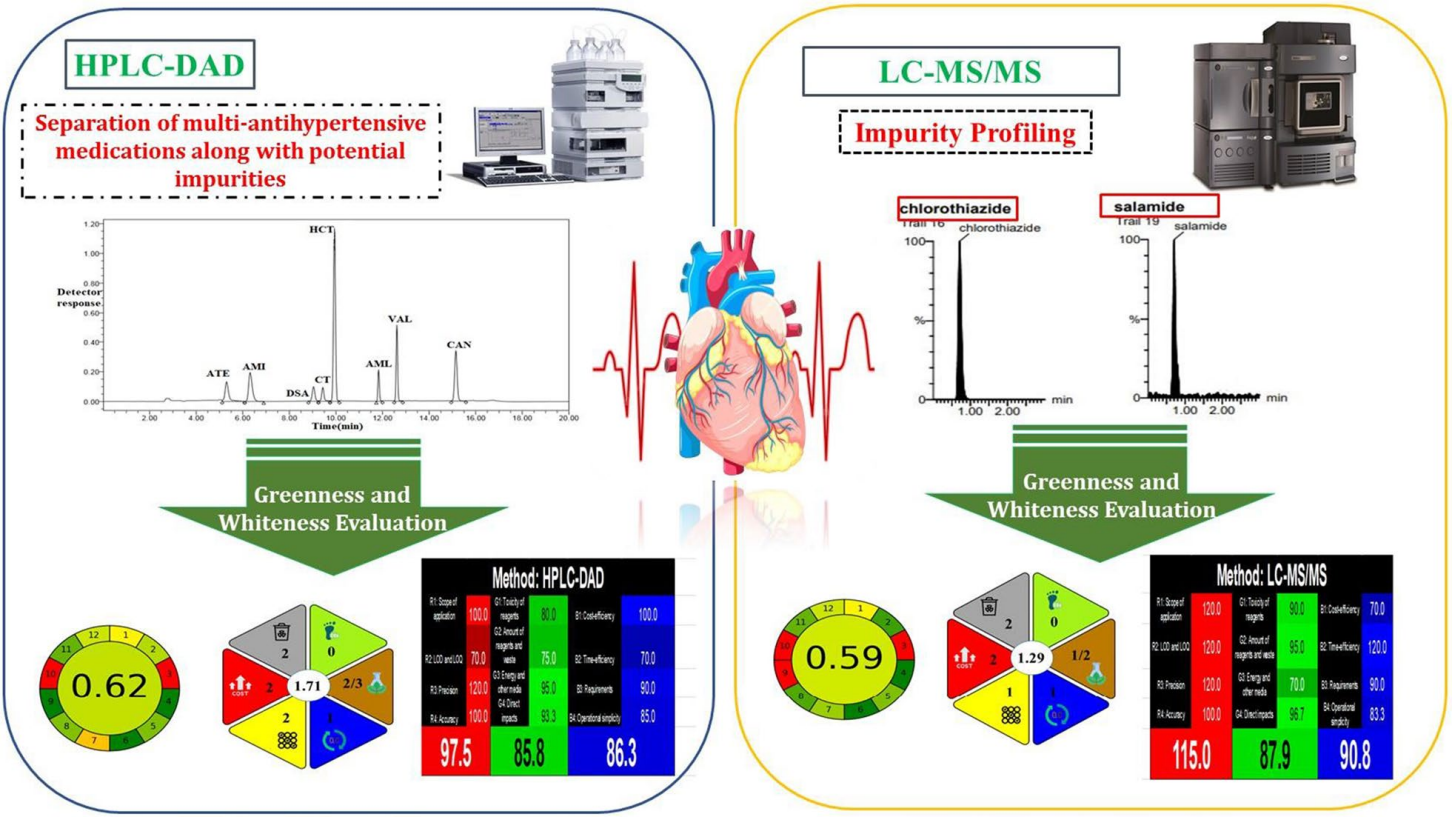

**Supplementary Information:**

The online version contains supplementary material available at 10.1186/s13065-023-01015-z.

## Introduction

Cardiovascular disease is considered one of the leading causes of morbidity and mortality worldwide. Cardiovascular disease is treated and managed by using combination of drugs where each drug functions by a different and unique mechanism of action to achieve and accomplish its therapeutic goals. Hypertension is a major worldwide problem affecting about 30% of world population [1]. It is the cause for more than 12.8% of total deaths annually [2]. Nevertheless, its treatment has been improved and enhanced significantly over the last two decades because of using multiple combination therapies.

Antihypertensive drugs’ additive effects are useful in the treatment of myocardial infarction, angina and arrhythmias and they can reduce blood pressure through blocking the action of the nervous system on the heart. Clinical sign verified that the combination of antihypertensive agents is very effective for the treatment of all previously mentioned diseases and has revealed to be superior to monotherapy than either agent alone. Fixed-dose combinations (multiple drugs in the same tablet) have additional benefits, including improved adherence by 24%, potentially reduced cost and easier indications. The drawback of such fixed-dose combinations is the inability to alter the dosage of just one of the drugs [3, 4].

Impurity profiling in modern pharmaceutical analysis has attained importance because of the undesirable, potentially toxic impurities which are hazardous to populations’ health. Controlling of impurities in active pharmaceutical ingredients and final formulated products is now getting from regulatory authorities a very important critical attention [5]. In the pharmaceutical field, an impurity is considered to be any organic/inorganic residual or material solvent other than the drug substances that rise out of synthesis, or any undesirable chemicals that remain and persist with the active pharmaceutical ingredients (APIs). Developing of the impurity may be originated either during formulation or upon APIs’ aging. Undesirable chemicals’ presence, even in small amounts, may affect the pharmaceutical products’ efficacy and safety [6]. Consequently, a widespread study of possible impurities as well as their detection and quantification in dosage forms, is definitely a vital and essential issue.

**Fig. 1:**
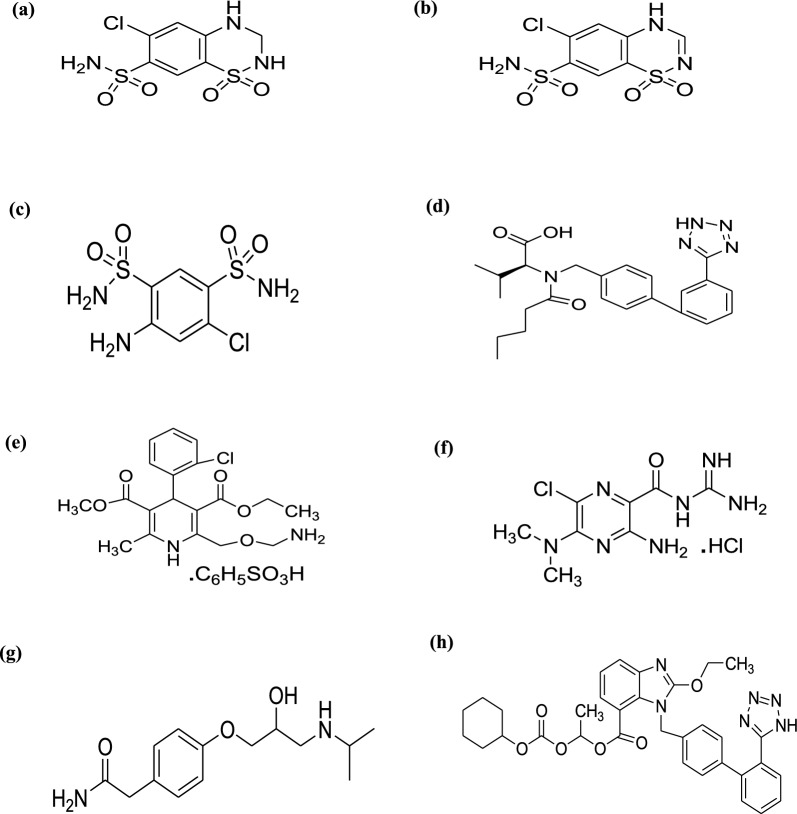
The chemical structures of **a** Hydrochlorothiazide (HCT), **b** Chlorothiazide (CT), **c** Salamide (DSA), **d** Valsartan (VAL), **e** Amlodipine besylate (AML), **f** Amiloride hydrochloride (AMI), **g** Atenolol (ATN), **h** Candesartan cilexetil (CAN)

Hydrochlorothiazide (HCT; Fig. 1a), is a thiazide diuretic used to manage and control hypertension. It is also effective in treating edema associated with moderate heart failure and with hepatic and renal maladies [7]. Chlorothiazide (CT; Fig. 1b) is documented in B.P [8] to be HCT process impurity A. It has lower pharmacological activity than HCT, possibly due to its incomplete absorption compared to HCT [9]. Salamide (DSA; Fig. 1c), is stated to be HCT process impurity B [8]^.^ In addition, DSA has been found to be a photolytic and hydrolytic degradation product of HCT [10, 11], moreover it has a chemical structure comprising a primary amino group, a functional group previously reported to be accompanied with carcinogenic activity [12, 13].

Valsartan (VAL, Fig. 1d), is considered as orally active non peptide triazole-derived antagonist of angiotensin II which has antihypertensive properties [14], that decreases the mortality in patients who have dysfunction in the left ventricle after myocardial infarction and utilized in the management and treatment of heart failure [7].

Amlodipine besylate (AML, Fig. 1e), is used to manage and treat hypertension and angina pectoris and it works by blocking the transmembrane influx of calcium ions into cardiac and vascular smooth muscles [7].

Atenolol (ATN; Fig. 1f), is considered as cardio-selective β1-selective adrenoreceptor antagonist. It causes decreasing in the heart rate and the heart muscle contraction’s force, it is utilized for managing and control of hypertension, arrhythmias and other cardiovascular diseases [7, 15].

Amiloride hydrochloride (AMI; Fig. 1g), exerts its effect via inhibiting the exchange of sodium–potassium ion via blocking the nephron’s distal renal tubule. This supports and enhances losing of sodium and water from the body and decrease potassium leakage [7].

Candesartan cilexetil (CAN; Fig. 1h), is a prodrug which undergoes hydrolysis to candesartan after administration via ester hydrolysis and it has an estimated bioavailability of 14% [16]. CAN is considered an angiotensin II receptor antagonist used alone or in combination with other drugs to treat and control high blood pressure [7].

The literature survey revealed several separation-based methods for resolving the studied antihypertensive drugs either in single or combined dosage forms. The mixture of HCT, VAL and AML has been recently analyzed by electrophoresis [17], high performance thin layer chromatography (HPTLC) [18–21], high performance liquid chromatography (HPLC) [22–26], LC–MS/MS [27–29] and ultra-performance liquid chromatography (UPLC) [30, 31] methods. While a mixture of HCT, ATN and AMI has been determined lately by capillary electrophoresis [32], HPTLC [33], and HPLC [34–36] methods. Furthermore, HCT mixture with CAN was simultaneously determined by HPTLC [37–39], HPLC [40–43], and LC–MS/MS [44–46] methods.

Due to the high sensitivity and specificity demonstrated, the high performance liquid chromatographic technique (HPLC) in combination with various detectors is recognized as the gold standard for the profiling of impurities in either pure active pharmaceutical ingredients (API) or in the ultimately formulated drug products [47, 48] and bioanalysis [49, 50]. Moreover, the magic privilege of combining HPLC with mass spectrometric detection, the detection here is according to the molecular mass of each substance, allows for tracing hundreds of biochemical, organic, and inorganic compounds in different matrices. Multiple reaction monitoring (MRM) is a highly specific and sensitive mass spectrometric technique that can selectively quantify compounds within complex mixtures. Only compounds that meet certain criteria, i.e. specific parent ion and specific daughter ions that match the mass of the target molecule are isolated within the mass spectrometer. The experiment becomes more sensitive while preserving the highest level of accuracy by disregarding any other ions that enter the mass spectrometer [51–57].

For the best of our knowledge, no previously reported method is found for the simultaneous determination of the six studied antihypertensive drugs (HCT, VAL, AML, ATN, AMI and CAN) in their combination and corresponding pharmaceutical formulations along with HCT British pharmacopoeia (BP) listed impurities in a single chromatographic run. So, the aim of this work is developing and validating accurate, sustainable, precise, specific, and robust HPLC–DAD method for simultaneous determination of six common antihypertensive medications and HCT impurities (CT and DSA) in diverse combinations containing HCT along with LC–MS/MS profiling of HCT potential and toxic impurities for the purpose of reducing quality control laboratory wastes, time, cost, and effort.

## Experimental

### Instruments and attached software

#### HPLC–DAD method

The HPLC system; Agilent 1200 Infinity series, Agilent Technologies, (Santa Clara, CA, USA) is operated by Agilent ChemStation software. This system is equipped with quaternary gradient pump (model G1311C), a photodiode array (model G4212B), an auto-sampler (model G1329B), a column oven (model G1316A), and degasser (Agilent). A pH-meter (Model 3510, Jenway, England) was utilized for recording and adjusting the pH of solvents.

#### LC–MS/MS method

The mass spectrometric analysis was performed using Waters Ultra Performance LC® system (Waters 3100 series, USA), including; binary solvent delivery system, autosampler, Waters Acquity TQD (Triple-Quad detector). Data was processed and acquired using Mass Lynx V4.1 software.

### Materials and reagents

Pure HCT (99.79 ± 0.65%), VAL (99.51 ± 0.55%), AML (99.74 ± 0.52%,) ATN (99.58 ± 0.69%) and AMI (99.61 ± 0.44%) samples were provided kindly by Egyptian International Pharmaceutical Industries (EIPICO), 10^th^ of Ramadan City, Sharqia Governorate, Egypt while CAN (99.58 ± 0.56%) was supplied by Pharaonia pharma, Borg Al Arab, Alexandaria, Egypt. Their purities were verified by applying official methods (RP-HPLC for HCT, VAL and AML while potentiometric titration method for ATN, AMI and CAN) [8, 58]. CT and DSA with certified purities of 99.60 and 99.80%, respectively, were purchased from Sigma Aldrich Chemie (Steinheim, Germany).

#### Pharmaceutical formulations

Exforge HCT® tablet (B.N. A518682), manufactured by Novartis, EL Amiria, and Cairo, and labeled to contain HCT 25.0 mg, VAL 160.0 mg and AML free-base 10.0 mg per tablet. Atenoretic® capsule (B.N. 71047), manufactured by Sigma Pharmaceutical Industries, Quesna Menoufia, and labeled to contain HCT 25.0 mg, ATN 50.0 mg and AMI 2.5 mg per capsule. Atacand Plus® tablet (B.N. 19015), manufactured by AstraZeneca, 6th of October, Giza, labeled to contain HCT 12.5 mg and CAN 16.0 mg per tablet.

Ethanol, methanol and acetonitrile of HPLC-grade (Sigma-Aldrich, Darmstadt-Germany). Potassium dihydrogen phosphate and orthophosphoric acid (El-Nasr Pharm. Co., Cairo, Egypt). Formic acid was supplied by Merck (Gernsheim, Germany). High purity distilled water was attained from "Aquatron" automatic water still A4000, Bibby Sterillin Ltd. (Staffordshire, England).

### Standard solutions preparation

#### HPLC–DAD method

Stock standard solutions (1.0 mg/mL) of HCT, VAL, AML, ATN, AMI and CAN and (0.1 mg/mL) of CT and DSA were prepared, separately, in 100.0 mL ethanol. Further dilution were made to obtain serial standard working solutions using the same solvent. Additionally, laboratory prepared solutions comprising different ratios of the eight analytes were attained by transferring different accurate aliquots from their corresponding stock and working solutions into a series of 10.0-mL volumetric flasks and the volumes were made up with the same solvent.

#### LC–MS/MS method

Stock standard solutions (1000.0 ng/mL) of CT and DSA were prepared, separately in 100.0-mL ethanol.

Storing of all previously prepared solutions was conducted in the refrigerator at 4.0–8.0 °C.

### Analytical methodology

#### Chromatographic and mass conditions

##### HPLC–DAD method

The chromatographic procedure was performed using Inertsil ODS-3 C_18_ column (250 × 4.6 mm, 5.0 μm), (Barcelona). Gradient elution programming was conducted using a mixture of solvent A (20.0 mM potassium dihydrogen phosphate, pH 3.0 ± 0.2, adjusted with phosphoric acid) and solvent B (acetonitrile), Table 1. The mobile phase components were filtered through 0.45 μm membrane filter and degassed before use in situ for 15.0 min usinic bath. The samples were also filtered by passing through membrane filter with 0.45 μm pore size before being automatically injected in 20.0 μL volumes and UV detected at 225.0 nm at room temperature.

**Table 1:** The HPLC–DAD gradient elution program

Time (min)	20.0 mM potassium dihydrogen phosphate solution, pH 3.0 ± 0.2 (%)	Acetonitrile (%)	Flow rate (mL/min)
0.00	90.0	10.0	1.0
3.00	90.0	10.0	1.0
10.00	50.0	50.0	1.0
10.01	20.0	80.0	1.5
20.00	20.0	80.0	1.5
20.10	90.0	10.0	1.5
25.00	90.0	10.0	1.5

##### LC–MS/MS method

Liquid chromatographic separations of CT and DSA were achieved using an Agilent Poroshell 120 EC-C_18_ column (4.6 × 50.0 mm, 2.7 µm) as a stationary phase and a binary isocratic mobile phase consisted of methanol and 0.1% formic acid (95:5, v/v). The operating flow rate through the column was 0.2 mL/min with a total 3.0 min run time. The sample injection volume was 10.0 µL. The selected-reaction monitoring (SRM) mode in the negative electrospray ionization was achieved for CT and DSA ions’ quantification and detection with the transition pairs at m/z 293.92 → 213.84 and 283.94 → 204.8, respectively. The gas/source dependent parameters were set as: collision energy 30.0 V and 25.0 V, and cone voltage, 45.0 V and 30.0 V for CT and DSA, respectively.

#### Linearity and plotting of calibration graphs

##### HPLC–DAD method

Into a set of 10.0-mL volumetric flasks, accurate aliquots were taken from respective analyte stock and working standard solutions and the volume was adjusted with ethanol. Linearity was examined across a concentration range of 0.1–100.0 µg/mL for HCT, VAL, AML and CAN, 0.05–100.0 µg/mL for ATN and AMI and 0.05–8.0 µg/mL for both CT and DSA. Triplicate 20 µL from the prepared samples were injected into the chromatographic apparatus applying the above mentioned chromatographic conditions. Calibration graphs were acquired by plotting relative peak area of each component (using 20.0 μg/mL of HCT, VAL, AML, ATN, AMI and CAN, and 2.0 μg/mL for CT and DSA as external standard) against their particular concentrations at 225.0 nm.

##### LC–MS/MS method

Calibration standards were made by accurately transferring various aliquots from CT and DSA standard solutions into 10.0-mL volumetric flasks with suitable dilution in ethanol, in order to cover the concentration range of 1.0–200.0 ng/mL and 5.0–200.0 ng/mL, respectively. In accordance with the aforementioned chromatographic conditions, 10.0 µL of the prepared solutions were subsequently analyzed in triplicates. Records of the chromatograms were made. Regression equations were then established for each component using calibration curves that relate average peak areas to the relevant concentrations.

#### Pharmaceutical dosage forms analysis procedure

Accurately weighing ten tablets/capsules, separately, of each pharmaceutical formulation, finely powdered and carefully mixed.

##### HPLC–DAD method

An accurate quantity equivalent to one tablet/capsule corresponding to 25/160/10 mg HCT/VAL/AML free-base, 25/50/2.5 mg HCT/ATN/AMI and 12.5/16 mg HCT/CAN was, separately, transferred to three 100-mL beakers. Afterwards, simple liquid extraction procedure with 50.0-mL ethanol was adopted, with continuous stirring for 10.0 min, filtered into three 100.0-mL volumetric flasks and subsequently diluted with ethanol to the mark. Afterwards, suitable dilutions of the prepared sample extracts were performed to reach the linearity ranges using ethanol and following the designated chromatographic conditions.

##### LC–MS/MS method

All aforementioned extraction steps were processed and appropriate dilution with ethanol was performed to the cited dosage forms to get solution with final claimed concentration of 50.0 μg/mL of HCT.

In order to confirm the accuracy of the suggested procedure in various pharmaceutical formulations, the standard addition technique was applied by adding small amounts of each component to the mixture extract.

## Results and discussion

### Analytical method development and optimization

The separation and quantitation of active pharmaceutical ingredients as well as the related impurities that have very similar structures, pose the biggest challenges in the development of analytical methods. During the experimental optimization cycle, several chromatographic conditions were attempted using one variable at time strategy.

#### HPLC–DAD method

Various chromatographic columns were tested to achieve optimal chromatographic separation and resolution of the eight compounds with sharp peaks such as Kinetex C_8_ (150.0 × 4.6 mm, 5.0 μm), Inertsil ODS-3 C_18_ column (250.0 × 4.6 mm, 5.0 μm), and CN column (150.0 × 4.6 mm, 3.0 μm). Utilizing Inertsil ODS-3 C_18_ column (250.0 × 4.6 mm, 5.0 μm) improved the separation and provided the best resolution for the analytes. In addition, it showed a better performance in the terms of theoretical plates. All experiments were carried out properly at room temperature.

Initially, isocratic elution utilizing various mobile phase compositions was attempted to resolve and separate all the studied components. Trials started using methanol–water followed by testing acetonitrile–water at various ratios. Acetonitrile showed promising results as an organic modifier compared to methanol, but neither system could achieve the desired separation. Then, different concentrations and pH of potassium dihydrogen phosphate solution as aqueous component were tried instead of water. Acceptable results obtained upon using 20.0 mM potassium dihydrogen phosphate, pH 3.0 ± 0.2, adjusted with phosphoric acid together with acetonitrile. Several ratios were tried, yet it was noticed that to obtain complete base-line separation for all structurally-related studied drugs within reasonable analysis time, gradient elution mode was applied. In addition, different flow rates were also checked along the experimental run to obtain the finest separation with minimum run time. The optimized gradient elution program is illustrated in Table 1.

Various UV detection wavelengths were examined like 210.0, 225.0, 230.0, 248.0 and 270.0 nm to achieve the highest possible sensitivity of the separated peaks. It was found that UV detection at 225.0 nm is suitable providing good sensitivity for quantification of all the cited compounds.

**Fig. 2:**
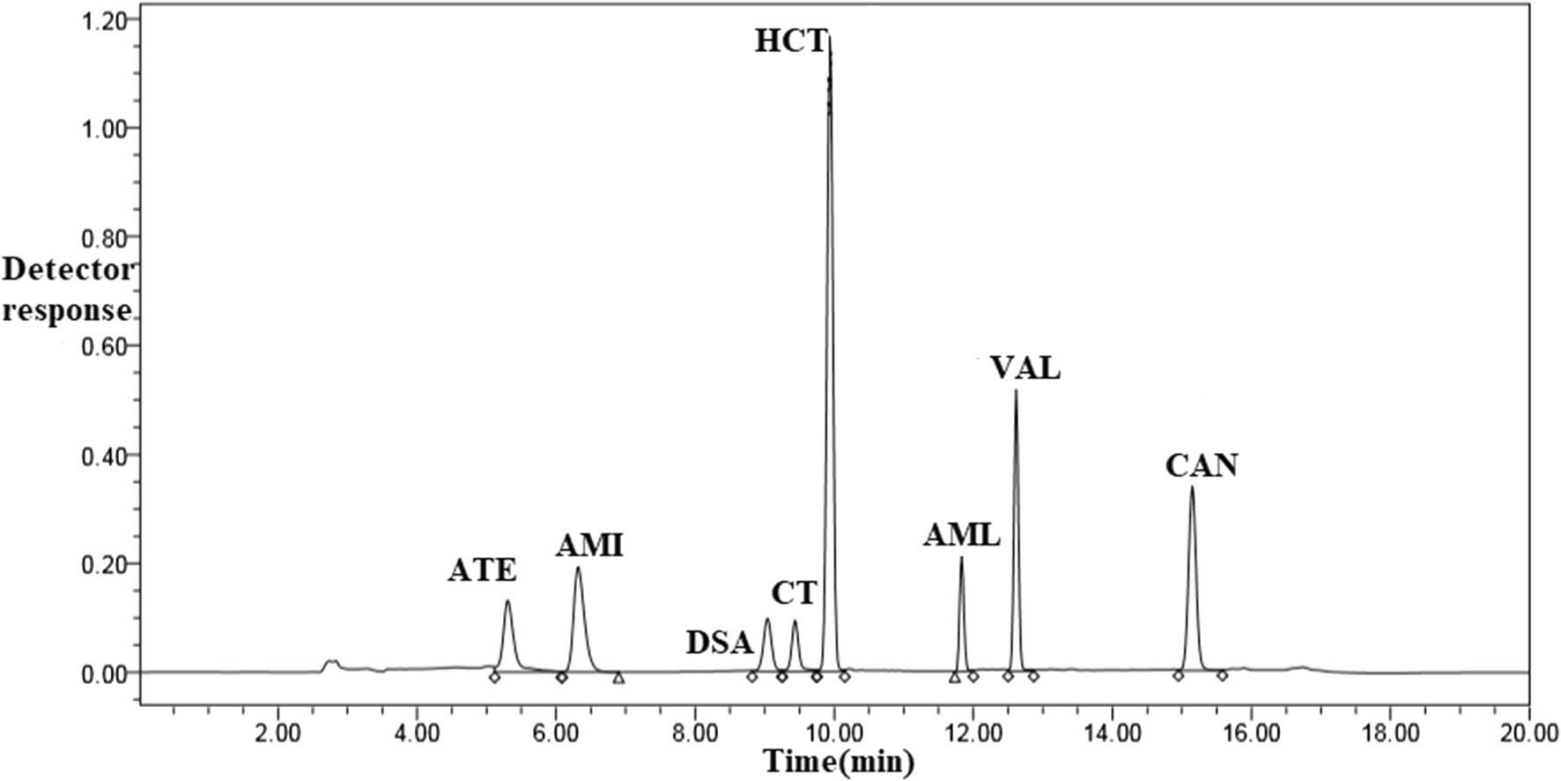
HPLC–DAD chromatogram of a mixture of 60.0 μg/mL VAL, AML, ATN, AMI, CAN and HCT and 5.0 μg/mL CT and DSA using gradient elution of 20.0 mM phosphate buffer adjusted to pH 3.0 ± 0.2 and acetonitrile at 225.0 nm

Finally, upon using the optimum chromatographic conditions, sharp symmetric peaks with satisfactory baseline separation of six antihypertensive medications along with HCT specified impurities (CT and DSA) was achieved within 15.0 min, as shown in Fig. 2. To investigate the operating system’s effectiveness for the analysis of the studied components, studying some selected parameters was performed such as symmetry factors, capacity, selectivity, and resolution in respect with USP guidelines [58]. The obtained values were within the acceptable limits and obeyed to reference values [59], verifying the performance and suitability of the chromatographic system for the intended use as shown in Table 2.

**Table 2:** System Suitability parameters of the proposed HPLC–DAD method

Parameter	ATN	AMI	DSA	CT	HCT	AML	VAL	CAN	Reference value [59]
Selectivity (α)	1.37	1.70	1.11	1.07	1.24	1.11	1.25		α > 1
Resolution (R_s_)	2.86	7.43	2.80	2.50	9.00	4.00	7.43		Rs > 1.5
Tailing factor (T)	1.15	1.23	0.95	1.06	0.97	1.07	0.98	1.14	T ≤ 2, T = 1 for symmetric peak
Column efficiency (N)	4806	3844	13766	36100	40000	55695	29122	23716	N > 2000
Height equivalent to theoretical plate (HETP) (cm/plate)	0.0052	0.0065	0.0018	0.0007	0.0006	0.0004	0.0009	0.0010	The smaller the value, the higher column efficiency
Retention time (T_R_) (min ± 0.2)	5.21	6.16	8.82	9.51	10.04	11.89	12.84	15.02	

#### LC–MS/MS method

**Fig. 3:**
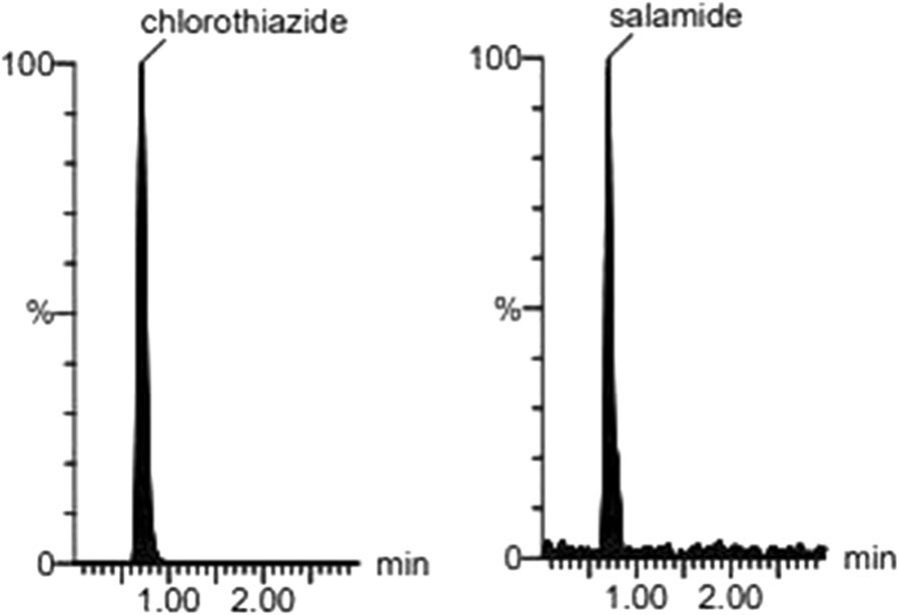
Selected reaction monitoring (SRM) chromatograms at LLOQ of standard CT and DSA, 1.0 ng/mL and 5.0 ng/mL, respectively

To attain optimized conditions of the proposed LC–MS/MS method for the sensitive quantitation of HCT impurities; CT and DSA, several chromatographic parameters were investigated. These parameters include; analytical stationary phase, organic modifier type, pH of the aqueous constituents and organic modifier–aqueous phase ratio. Optimization of these parameters was conducted based on the intensity and shape of the peak. The use of Agilent Poroshell 120 EC-C_18_ column (50 × 4.6 mm, 2.7 µm) has greatly improved the peak’s symmetry. Several experiments were conducted utilizing different proportions of either methanol or acetonitrile as the organic phase with water, aqueous formic acid or acetic acid (0.1–0.5%). The mobile phase consisting of methanol—0.1% formic acid (95:5, v/v) pumped isocratically at 0.2 mL/min at room temperature gave higher detection sensitivity for both CT and DSA with fast (run time 3.0 min) and reliable separation, Fig. 3.

LC–MS/MS with mode of selected reaction monitoring, affords sensitivity and selectivity necessities for analytical methods utilized for the detection of very low concentrations of the pharmaceutical impurities. The parameters of the mass spectrometric were optimized to maximize the response of each of the transition of the precursor/product. The SRM was attained in the negative ion mode. The most sensitive mass transitions for CT and DSA were found to be m/z 293.92 → 213.84 and 283.94 → 204.8, respectively, Additional file 1: Fig. S1.

### Validation parameters of the proposed analytical methods

The suggested chromatographic methods’ validation has been carried out in accordance with ICH guidelines [60].

#### Linearity

Injection of at least six samples within the entire working ranges was performed into the HPLC system using the aforementioned chromatographic conditions in triplicates.

##### HPLC–DAD method

The assay’s calibration curve was in the concentration range of 0.1–100.0 μg/mL of HCT, VAL, AML and CAN, 0.05–100.0 μg/mL of ATN and AMI, 0.05–8.0 μg/mL of CT and DSA. Construction of calibration curve of each drug was carried out representing the relationship between the peak area ratios (using external standard technique) and the corresponding concentrations of each drug in μg/mL and the computation of regression equations was performed, Table 3.

**Table 3:** Validation and Regression parameters of the proposed HPLC–DAD and LC–MS/MS methods

Method Parameter	HPLC–DAD method								LC–MS/MS method	
	HCT	CT	DSA	VAL	AML	ATN	AMI	CAN	CT	SAL
Linearity range^a^	0.10–100.00	0.05–8.00		0.10–100.0		0.05–100.00		0.10–100.00	1.0–200.0	5.0–200.0
Regression equation parameters										
Slope	0.0530	0.4789	0.4957	0.0449	0.0493	0.0492	0.4940	0.0502	92.062	1.4412
Intercept	0.0051	0.0343	0.0134	0.0889	0.0169	0.0084	0.0155	0.0020	29.337	0.8209
Correlation Coefficient (r)	1.0000	1.0000	1.0000	1.0000	1.0000	1.0000	1.0000	1.0000	0.9999	0.9999
Accuracy ^b^ (Mean ± SD)	99.51 ± 0.68	100.44 ± 0.59	100.52 ± 0.69	100.18 ± 0.86	100.57 ± 1.05	99.25 ± 0.77	99.97 ± 0.69	99.03 ± 0.75	99.92 ± 0.60	99.64 ± 0.77
Precision (± %RSD)										
Repeatability ^c^	0.85	0.38	0.69	0.75	0.48	0.37	0.25	0.55	0.88	0.61
Intermediate precision ^d^	0.91	0.80	0.86	0.87	0.53	0.48	0.48	0.76	1.00	0.78
LOD ^e^	0.01	0.01	0.01	0.02	0.01	0.01	0.01	0.03	0.19	0.14
LOQ ^e^	0.05	0.02	0.02	0.06	0.02	0.03	0.03	0.08	0.59	0.41
Robustness ^f^	0.96	0.79	0.88	0.79	0.88	1.15	0.85	0.98	0.65	0.52

^a^ For HPLC–DAD method: in μg/mL and for LC–MS/MS method: in ng/mL

^b^ Accuracy was checked using concentrations for HPLC–DAD method: 0.2, 40.0 and 80.0 µg/mL for HCT, VAL, AML, ATN, AMI and CAN and 0.3, 3.0 and 7.0 µg/mL for CT and SAL while for LC–MS/MS method: 40.0, 80.0 and 120.0 ng/mL for CT and SAL

^**c**^ and ^d^ are repeatability and intermediate precision, respectively (n = 9) percentage relative standard deviation of three different concentrations in triplicate for HPLC–DAD method: 0.5, 20.0 and 60.0 µg/mL for HCT, VAL, AML, ATN, AMI and CAN and 0.1, 2.0 and 5.0 µg/mL for CT and SAL while for LC–MS/MS method: 5.0, 50.0 and 150.0 ng/mLfor CT and 10.0, 50.0 and 150.0 ng/mL for SAL

^e^ LOD and LOQ were calculated as per ICH, 3.3 × SD of the intercept/slope and 10 × SD of the intercept/slope, respectively

^f^ For HPLC–DAD: average %RSD of the change in detection wavelength (± 1 nm) and flow rate (± 0.1 mL/min), while for LC–MS/MS: average %RSD of the change in the mobile phase composition (± 2%)

##### LC–MS/MS method

CT and DSA assay was calibrated in the range of 1.0–200.0 ng/mL and 5.0–200.0 ng/mL, in order. Linearity was evaluated and regression equations were computed by generating calibration curves relating the average peak areas to the corresponding concentration of each component, Table 3.

#### Accuracy

The result’s accuracy was investigated and examined by applying the proposed methods for determination of various blind samples containing different proportions of studied components. Acceptable percentages recoveries were obtained after analyzing three concentration levels through the developed linearity range for the studied drugs, Table 3, ensuring good accuracy of the developed analytical methods.

#### Precision

To evaluate precision of the developed methods, injecting sample solutions containing the targeted analytes three times within one day, at three different concentration levels, intra-day variations (repeatability) was evaluated. Whereas, by examining nine samples (3 replicates of 3 concentration levels) over the course of three days, inter-day variations (intermediate precision) was assessed. The results obtained were presented as a percentage of the relative standard deviation (%RSD) of the peak responses. The calculated (%RSD) indicated precision of studied methods and their applicability for quality control analysis of the cited components, Table 3.

#### Specificity

The developed method’s specificity was checked both qualitatively and quantitatively. This was achieved via analyzing diverse laboratory prepared mixtures comprising the studied compounds in different composition ratios within the linearity ranges and pharmaceutical formulation to test for the possible interference arising from tablets additives. The obtained results were represented in the form of mean % recovery ± SD. The results obtained were acceptable and within the standard limits, assuring specificity of the method and no significant interference from common industrial excipients, Additional file 1: Table S1.

#### Limits of detection and quantitation (LOD and LOQ)

The LOD and LOQ were estimated based on the slope and standard deviation of response in accordance with ICH standards using the following equations; 3.3 × σ/S and 10 × σ/S, in order. Where, σ is the regression line's y-intercept standard deviation and S is the slope of calibration plots. Results of testing LOD and LOQ values are given in Table 3. These demonstrated that the devised LC–MS/MS approach could detect and quantify HCT impurities with high sensitivity and below the specified pharmacopoeial limits.

#### Robustness

Here, experimental conditions of the proposed methodologies were slightly and deliberately altered. For evaluation of robustness of HPLC–DAD method, small change in detection wavelength (± 1.0 nm), or flow rate (± 0.1 mL/min) was performed. Yet, for LC–MS/MS method varying mobile phase composition (± 2.0%) was tested. While studying each previously mentioned factor, other factors were kept constant. Afterwards, recording the response together with important parameters that could affect the resolution between the studied antihypertensive drugs was performed. Robustness is expressed as %RSD of response and results are shown in Table 3, ensuring sufficient robustness of the established chromatographic methods.

### Application of the proposed chromatographic methods on pharmaceutical formulations

The proposed HPLC–DAD is valid and applicable for determination of the six antihypertensive drugs in their pharmaceutical formulations (Exforge HCT®, Atenoretic® and Atacand plus®) without interference from the excipients. The results are in good agreement with their nominal content as shown in Table 4. This confirms the appropriateness of the proposed chromatographic method for the routine analysis of previously mentioned analytes in their combined formulations. The validity of the developed method is further assessed by implementing the standard addition procedure, revealing no excipients interference. The results attained are revealed in Table 4.

**Table 4:** Determination of the studied drugs in pharmaceutical dosage forms by the proposed HPLC–DAD method and application of standard addition technique

Pharmaceutical dosage form	HCT	VAL	AML	ATN	AMI	CAN
Exforge HCT^®^ Tablets, B.N. A518682, Each tablet is labelled to contain 25.0 mg HCT, 160.0 mg VAL & 10.0 mg AML						
% Found ± SD ^a^	99.73 ± 0.88	99.47 ± 0.59	99.85 ± 0.56	–	–	–
Standard addition ^b^ % recovery of the pure added ± SD ^c^	98.45 ± 0.77	99.98 ± 0.37	99.31 ± 0.30	–	–	–
Atenoretic® Tablets, B.N.71047, Each tablet is labelled to contain 25.0 mg HCT, 50.0mg ATN & 2.5 mg AMI						
% Found ± SD ^a^	99.66 ± 0.77		–	100.95 ± 0.25	99.13 ± 0.65	–
Standard addition ^b^ % recovery of the pure added ± SD ^d^	100.54 ± 0.52	–	–	99.83 ± 1.03	99.41 ± 0.69	–
Atacand plus® Tablets, B.N. 19015, Each tablet is labelled to contain 12.5 mg HCT, & 16.0 mg CAN						
% Found ± SD ^a^	99.23 ± 0.57	–	–	–	–	98.80 ± 0.94
Standard addition ^b^ % recovery of the pure added ± SD ^e^	98.73 ± 1.04	–	–	–	–	98.70 ± 0.68

^a^ Average of three experiments

^b^ Average of three experiments

^c^ Claimed Concentration; 32 µg/mL VAL, 1 µg/mL AML and 2.5 µg/mL HCT and pure added equivalent to 8.0, 16.0, 32.0 µg/mL VAL, 0.5, 1.0, 2.0 µg/mL AML and 1.0, 2.5, 5.0 µg/mL HCT

^d^ Claimed Concentration; 1.25 µg/mL AML, 25 µg/mL ATN and 12.5 µg/mL HCT and pure added equivalent to 0.5, 1.25,2.5 µg/mL AML,12.5, 25.0, 50.0 µg/mL ATN and 6.0, 12.5, 25.0 µg/mL HCT

^e^ Claimed Concentration; 16 µg/mL CAN and 12.5 µg/mL HCT and pure added equivalent to8.0, 16.0, 32.0 µg/mL CAN and 6.0, 12.5, 25.0 µg/mL HCT

Regarding LC–MS/MS method, in order to remove the interference from possible co-extractives that could affect mass ionization [61], standard addition method (SAM) was applied for the quantification of CT and DSA in the three studied dosage forms [62]. The studied dosage forms were enriched with CT and DSA at three concentration levels and signal intensities of the fortified samples were measured. A linear line was obtained from a calibration plot of the peak areas versus corresponding standard added of CT and DSA, separately, to the spiked sample containing unknown concentrations of them, Additional file 1: Fig. S2. The unknown concentration of both CT and DSA were calculated from the respective point (x-axis) whereupon the extrapolated linear line intersects the concentration axis at zero response, Additional file 1: Fig. S2. As per the British Pharmacopoeia [8], HCT specified impurities’ limit in pharmaceutical formulations should not exceed 0.1% of labeled amount. It was found that both CT and DSA were detected in all of the studied dosage forms and their levels were below the defined permissible limits (less than 0.1% w/w of HCT in a tablet), Table 5, thereby indicating that all impurities are well controlled. As a result, the suggested LC–MS/MS method satisfies the sensitivity and selectivity requirements for analytical techniques used to spot and detect pharmaceutical impurities at incredibly low concentrations.

**Table 5:** Determination of the studied drugs in pharmaceutical dosage forms by the proposed HPLC–DAD method and application of standard addition technique

Pharmaceutical Formulation	CT			DSA		
	Found (ng/mL)	Standard added ^a^ (ng/mL)	%R of standard added ^b^	Found (ng/mL)	Standard added ^a^ (ng/mL)	%R of standard added ^b^
Exforge HCT^®^	1.07	25.0 (0.05%)	98.61	0.08	25.0 (0.05%)	101.01
Tablets		50.0 (0.1%)	101.04		50.0 (0.1%)	100.02
(Labeled to contain HCT 25.0 mg, VAL 160.0 mg and AML 10.0 mg)		100.0 (0.2%)	99.83		100.0 (0.2%)	100.20
B.N. (A518682)		Mean ± SD	99.83 ± 1.21		Mean ± SD	100.41 ± 0.53
Atenoretic^®^	0.64	25.0 (0.05%)	100.94	1.47	25.0 (0.05%)	100.37
capsules		50.0 (0.1%)	99.30		50.0 (0.1%)	100.17
(Labeled to contain HCT 25.0 mg, ATN 50.0 mg and AMI 2.5 mg)		100.0 (0.2%)	100.12		100.0 (0.2%)	100.08
B.N. (71047)		Mean ± SD	100.12 ± 0.82		Mean ± SD	100.21 ± 0.15
Atacand Plus®	1.03	25.0 (0.05%)	101.56	0.05	25.0 (0.05%)	99.88
Tablets		50.0 (0.1%)	98.83		50.0 (0.1%)	100.10
(Labeled to contain HCT 12.5 mg and CAN 16.0)		100.0 (0.2%)	100.19		100.0 (0.2%)	99.99
B.N. (19015)		Mean ± SD	100.19 ± 1.36		Mean ± SD	99.99 ± 0.11

^a^ Percentage w/w from HCT in a tablet

^b^ Average of three determinations

### The proposed method’s greenness evaluation

The conformity of the offered method to the assumptions of green analytical chemistry was documented and indexed across many evaluation tools in order to figure out a clear image of the presented method’s greenness profile. These utilized tools were, analytical greenness metric (AGREE) method [63] and HEXAGON method [64].

#### Greenness analytical tool (AGREE)

Pena-Pereira created an innovative, printable greenness evaluation AGREE software in 2020 [63] which operates using a calculator that may be downloaded from this link (https://mostwiedzy.pl/AGREE). AGREE metric incorporates the twelve green analytical chemistry theories, twelve sections make up the automatically generated pictogram, and each one has a distinct color spectrum between dark green (= 1) until dark red (= 0). The pictogram's center contains the overall score as a fraction of unity ranging from zero to one. The color combination displaying the output of the 12 AGREE pictogram sections is the result producing color in the center. The best strategy yields a score of 1, using the color dark green. The pictograms in Table 6 shows how well-developed HPLC–DAD and LC–MS/MS approaches capture greenness. While LC–MS/MS method employed for the assessment of CT and DSA had good greenness scores 0.59, HPLC–DAD method had a score of 0.62 with higher green performance due to consuming less energy. Furthermore, AGREE software provided comprehensive information about the entire analytical process related to each principle in green chemistry in a form of pdf file, by highlighting the analytical procedures' weakest parts that require additional greenness adjustment. For the previously chromatographic procedures, comprehensive reports with colorful pictograms and highlighted reports were shown in Additional file 1: Figs. S3 and S4.

**Table 6:** AGREE, HEXAGON and WAC profiles created for the suggested HPLC–DAD and LC–MS/MS

	HPLC–DAD	LC–MS/MS
AGREE		
HEXAGON		
WAC		

In conclusion, AGREE metrics provided us with good information about the greenness of the chromatographic methods. Concerning to what was mentioned previously, it is noted that this tool is easy for the analyzer to extract data related to the greenness of the used analytical method, but it lacks information about the validity of this method and its analytical efficiency, as this tool did not provide information showing that the chromatographic method with tandem mass spectrometry was of higher sensitivity than the chromatographic method with diode array detector. In order to present a comprehensive and complete green file on the used methods, the efficiency and validity results of the analytical methods must be taken into account. Therefore, other green metrics were used.

#### The HEXAGON assessment tool

Modern multi-criteria tools that aim to completely evaluate analytical methodologies include the HEXAGON tool [64]. The examined factors include analytical performance, sustainable development, environmental friendliness, and financial cost. They are investigated using the penalization idea. A hexagonal pictogram that represents the total assessment enables comparison of various analytical techniques. The following factors were assessed:

1. The quality parameters were divided into two groups by the figures of merit FM: the sample processing, procedure characteristic, and calibration process were all included in the FM-1. For the accuracy and quality control of the analytical approach, FM-2 were used.
2. The toxicity and safety triangle demonstrated the danger and toxicity of the chemical items used, as well as the analyst's safety.
3. Residue's triangle, where the amount of waste produced and its treatment were assessed.
4. To assess the effects on the environment, a carbon footprint triangle was used.
5. Yearly economic cost of the analytical method within study was calculated taking in consideration the cost of the reagents, materials, electricity used, and the staff needed to conduct the analytical determination.

Penalty points (PP), which convert into a final score ranging from 0 to 4, were used to measure deviation from idealistic for each of the evaluation factors. You can find more information related to the evaluating tables and PP ranges in the original article [64]. Six symmetrical triangles connected to the information mentioned are grouped together in a hexagon diagram. Each triangle has a color that corresponds to a criterion rather than a score ranged from 0 to 4. The most green and sustainable method is the one with maximum zeros. In order to compare the different analytical approaches from a single dataset, the mathematical average (mean) of the previously six mentioned triangles with the 0–4 range is computed and drawn in the center of the hexagon, and the scale is related to the examined analytical method's performance to the scores of 0, 1, 2, 3, and 4, respectively, for perfect, good, suitable, fair and weak [65]. The mathematical averages of the hexagon scores (mean) for the HPLC–DAD and LC–MS/MS, were 1.71 and 1.29.

According to the previously mentioned scores, both HPLC–DAD & LC–MS/MS are considered as good green analytical methods, results from the HEXAGON tool confirmed that two chromatographic procedures were performed as intended. Both methods achieved the same values in each triangle, except for the toxicity/safety triangle and analytical figures of merit FM1 triangle which had different results.

With a 1/2 score in the toxicity and safety triangle, the LC–MS/MS approach is in fact superior to the HPLC–DAD methods. In LC–MS/MS, the solvent employed (ethanol and formic acid) is the major cause of fluctuation in this parameter. However, in HPLC–DAD, the separation was achieved using some hazardous chemical reagents: potassium dihydrogen phosphate, phosphoric acid, acetonitrile, and ethanol and hence were given a value of 3/2.

With 2 score in the FM1 triangle, due to the working range of concentrations, required long duration, and lower limit of detection, HPLC–DAD exhibits weaker adaptation to the figures of merit for the calibration technique than LC–MS/MS which raised the score 1 for FM1 triangle.

The analytical advantages of LC–MS/MS over HPLC–DAD include a wider linear dynamic range as well as improved lower detection limits.

As for the carbon footprint measures, which are taken into consideration, the energy usage of the equipment used and the time it takes to conduct the study, also quantify the environmental impact. It is commonly known that HPLC with a diode array detector significantly uses less energy than HPLC equipment with a tandem mass spectrometry. As a result, the carbon footprint measurement and the annual cost for the LC–MS/MS method is higher than the measurement for the HPLC–DAD method, and the HPLC–DAD approach incorporates a more environmentally friendly process.

**Fig. 4:**
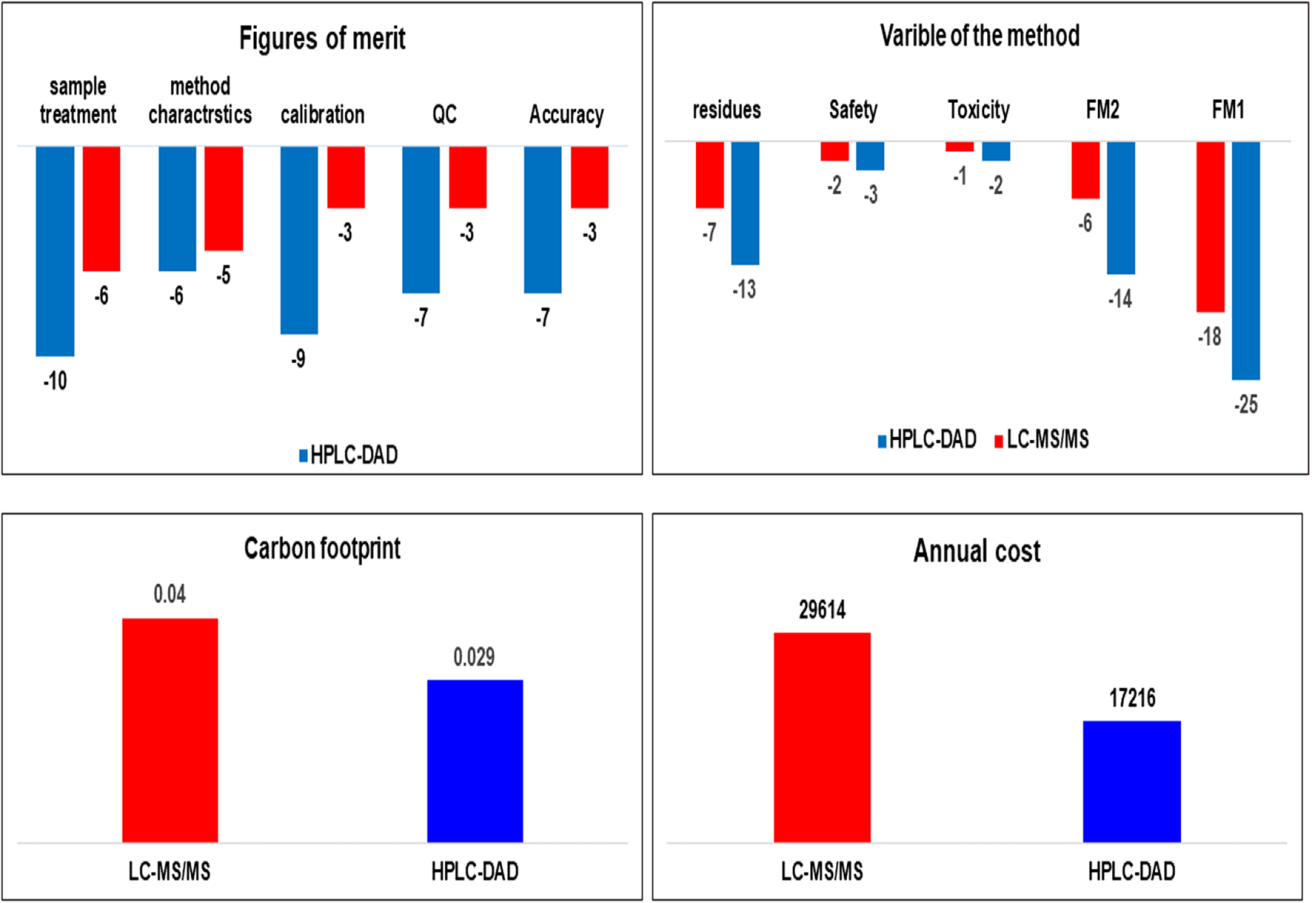
Assessment of penalty points for the figures of merit, variable of the methods, carbon footprint in kilograms of CO_2_ equivalent, and annual economic cost related to the suggested HPLC–DAD & LC–MS/MS methods

The hexagon pictogram greenness evaluation of the HPLC–DAD and LC–MS/MS techniques as well as the diagrams representing the penalty points of the FM, variable of the method, the carbon footprint and the annual cost are depicted in Table 6 and Fig. 4 describing the results from the HEXAGON evaluation of the HPLC–DAD and LC–MS/MS techniques. More information related to HEXAGON tool reported in Additional file 1: Fig. S5 and Table S2.

Further explanation related to the adapted chromatographic methods with different detectors DAD diode array detector and tandem mass spectrometry MS/MS which were used in this paper to separate six active antihypertensive drugs (HCT, VAL, AML, ATN, AMI and CAN), as well as the two listed impurities (CT & DSA) presented with minimum concentration should be clarified.

The reader may wonder how the LC–MS/MS method got higher points than the HPLC–DAD method when using the HEXAGON tool to assess greenness, while the result was opposite in the AGREE tool.

This difference can be explained simply; the HEXAGON tool takes into account several criteria to evaluate the greenness of the method, including: the efficiency of the analytical method, its sensitivity, the amount of electrical energy consumed, the amount of solvent consumed, and the time to complete the analysis. While the AGREE method does not include all the criteria mentioned above, but only covers criteria that comply with the twelve greening principles, making the HEXAGON tool more inclusive and objective.

We must point out to the difficulty of applying this HEXAGON tool because there is no pre-programmed Excel worksheet or automated program available, which requires a great effort from the researcher applying this method.

### White analytical chemistry (WAC)

White Analytical Chemistry method has been updated in 2021 by Nowak et al. [66]. The purpose of the WAC is to offer a unique ideology tool for applying sustainable development concepts in analytical chemistry. It is important to keep in mind that the sustainable development concept, which is currently being globally enhanced, is in reality multidimensional. i.e., it emphasizes the importance of attempting to achieve a balance between the validity of the research, which is correlated with the development of science, and the protection of the environment.

This metric would allow one to articulate all major expectations for desired technique characteristics in a formal and systematic manner, including green and other remaining requirements. Three complementing sections make up these pillars. Each section has a different color (red, green, blue) and involves four specific criteria that assess essential concepts of the analytical method, then by mixing the previously mentioned color, the white color of the method will be obtained.

Red section assesses the analytical effectiveness through four algorithms involving: R1 for application scope, LOD and LOQ in R2, R3 for accuracy and R4 for Precision.

Green section assesses the green environmental impact through four algorithm G1 for reagent toxicity, G2 for the quantity of wastes and reagents, energy and other media sorted in algorithm G3, and the direct effects on people, animals, and genetic naturalness are covered in G4 algorithm.

Blue section which makes sense in light of their significant influence on practical usefulness and economic conditions through four algorithms, B1 covers the cost effectiveness, B2 deals with the time efficiency, B3 for the requirements of the method, and B4 deals with the simplicity of operation. WAC tool also named as (RGB 12) in relation to the number of the previously mentioned rules.

The WAC definition describes "white" as an analytical approach which is well matched and purpose-fit. The suggested chromatographic techniques were examined and impartially contrasted with one another. Results of this methods' evaluation utilizing the WAC tool are reported in Table 6.

The suggested HPLC–DAD method had the highest values in the LOD and LOQ whereas the proposed LC–MS/MS method had the lowest value in LOD and the highest scope of application for the validation criterion (red band). As long as the two suggested methods adhere to the ICH guidelines, they are all accurate and precise.

Studying environmental factors (green band) revealed that the greenness assessment results for both methods are close, this can be explained as follows: HPLC–DAD method used more solvents and the analysis time was 16 min, but this method consumes less energy, while in the LC–MS/MS method the number of solvents was less and the analysis time is low only 3 min, but this method consumes a lot of energy. None of the techniques examined used either animals or genetically altered organisms (GMO).

**Fig. 5:**
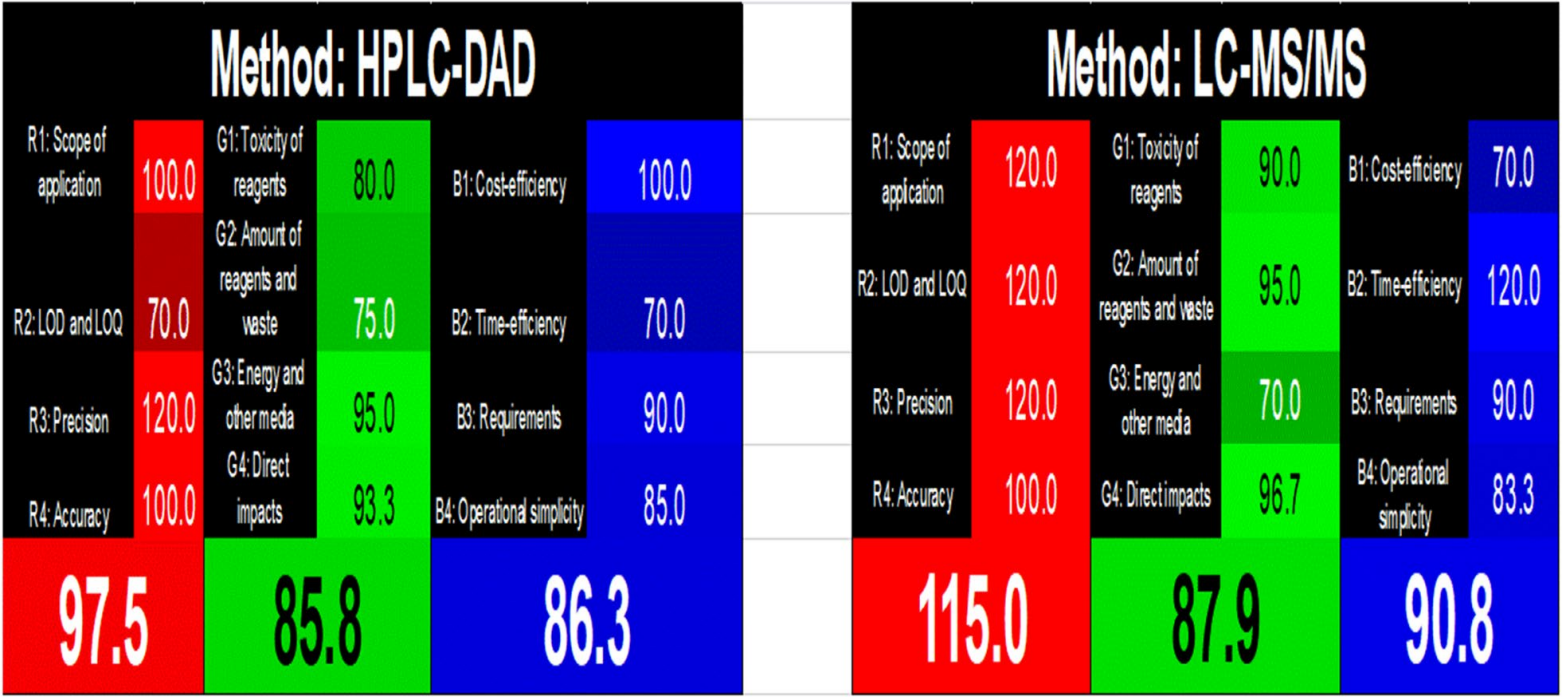
Display of the outcomes of the two chromatographic approaches HPLC–DAD & LC–MS/MS using the RGB12 tool

In terms of productivity and sustainability (blue band), HPLC–DAD and LC–MS/MS were both somewhat affordable, time-efficient, and simple to use. The measurements and laboratory work were supposed to be completed in the same lab according to the protocols, therefore there was no need for extra transportation or analysis equipment. The values of the arithmetic means for the three bands, R(%), G(%), and B(%), are shown individually in the Fig. 5. The findings of this whiteness investigation were illustrated in Additional file 1: Fig. S6 (in the Supplementary File). Overall, the scores 97.9% indicated that the suggested LC–MS/MS is superior to the reported HPLC–DAD, which is ranked second with a score of 89.9%.

We note that the results of the greenness and sustainability evaluation in the WAC tool are compatible with the results of the HEXAGON tool and inconsistent with the AGREE tool, this is due to the fact that the WAC and HEXAGON tools take the results concerning to the validity of the method into consideration, while the procedure of the AGREE tool depends only on the evaluation according to the twelve greenness principles.

## Conclusion

Novel, robust and sustainable HPLC–DAD and LC–MS/MS methods have been developed and fully optimized for the concurrent determination of six commonly prescribed antihypertensive drugs in bulk and pharmaceutical dosage forms and profiling of HCT potential impurities (CT and DSA). The developed methodologies are straightforward, sensitive and accurate with high efficiencies. Moreover, they were validated according to ICH guidelines and demonstrated their ability to assay the studied drugs as well as quantitative analysis of impurities at low level and consequently can be utilized in routine analysis of the proposed drugs in their bulk and diverse pharmaceutical formulations. Three different tools for assessing and evaluating the greenness and whiteness of the analytical chromatographic approaches were successfully applied. The 12 GAC concepts are best covered by the AGREE tool, which is also the most automated, easily, free download and user-friendly instrument. The HEXAGON tool is the most objective multi-criteria tool by employing the penalization methodology for scoring, besides hexagon pictogram scores generated by the HEXAGON tool in addition to the arithmetic mean computation allow to easily compare the chromatographic approaches by simple visual inspection. The WAC tool is widely regarded as an innovative tool for assessing sustainability and greenness using a simple, free Excel spreadsheet. The evaluation and validation tests listed above found positive results. All of the aforementioned assessment green tools supported the application of the HPLC–DAD and LC–MS/MS techniques in pharmaceutical quality control units.

### Supplementary Information

**Additional file 1: Figure S1.** Representative mass spectra at negative mode ([M-H]) of a CT and (b) DSA. **Figure S2.** Standard addition plots for determination of a CT and b DSA, in a Exforge HCT® Tablets, b Atenoretic® capsules and c Atacand Plus® Tablets. **Figure S3.** AGREE report with detailed scores for HPLC-DAD method obtained from AGREE software. **Figure S4.** AGREE report with detailed scores for LC-MS/MS method obtained from AGREE software. **Figure S5.** Hexagon results for HPLC-DAD and LC-MS/MS methods and the mean average in the middle circle. **Figure S6.** The white line represents 100%, which means that the major assessment results from the WAC analysis are fully appropriate for the intended use. The values above 100 suggest the presence of additional capabilities above what is currently required. **Table S1.** Determination of the proposed drugs in the laboratory-prepared mixtures by the proposed HPLC-DAD method. **Table S2.** Total results of the parameters for the suggested approaches, according to the ranges of penalty points for the HEXAGON evaluation tool.

## Data Availability

All data generated or analysed during this study are included in this published article.
