# Supplementary material for: Sustainable chromatographic quantitation of multi-antihypertensive medications: application on diverse combinations containing hydrochlorothiazide along with LC–MS/MS profiling of potential impurities: greenness and whiteness evaluation
Source: BMC Chem. 2023 Aug 19;17(1):101. doi: 10.1186/s13065-023-01015-z (PMC10439576; doi:10.1186/s13065-023-01015-z)
Supplement: Supplementary file 1 — Additional file 1: Figure S1. Representative mass spectra at negative mode ([M-H]) of a CT and (b) DSA. Figure S2. Standard addition plots for determination of a CT and b DSA, in a Exforge HCT® Tablets, b Atenoretic® capsules and c Atacand Plus® Tablets. Figure S3. AGREE report with detailed scores for HPLC-DAD method obtained from AGREE software. Figure S4. AGREE report with detailed scores for LC-MS/MS method obtained from AGREE software. Figure S5. Hexagon results for HPLC-DAD and LC-MS/MS methods and the mean average in the middle circle. Figure S6. The white line represents 100%, which means that the major assessment results from the WAC analysis are fully appropriate for the intended use. The values above 100 suggest the presence of additional capabilities above what is currently required. Table S1. Determination of the proposed drugs in the laboratory-prepared mixtures by the proposed HPLC-DAD method. Table S2. Total results of the parameters for the suggested approaches, according to the ranges of penalty points for the HEXAGON evaluation tool. [file 13065_2023_1015_MOESM1_ESM.docx]

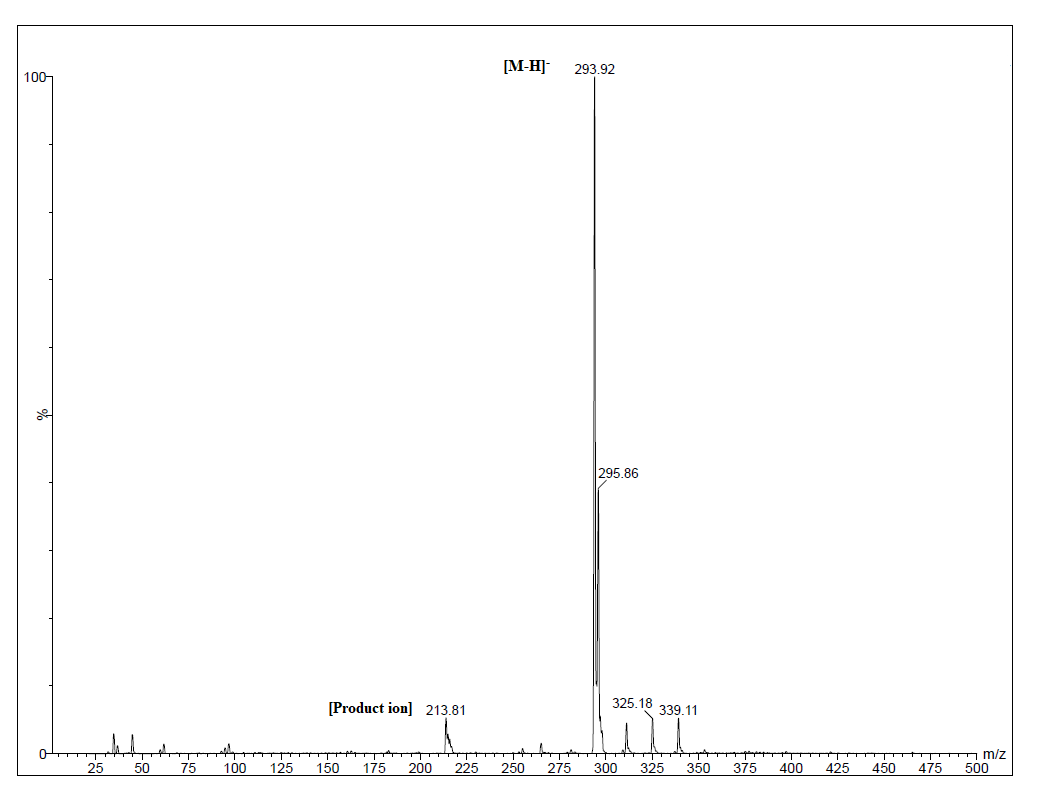


**(a)**


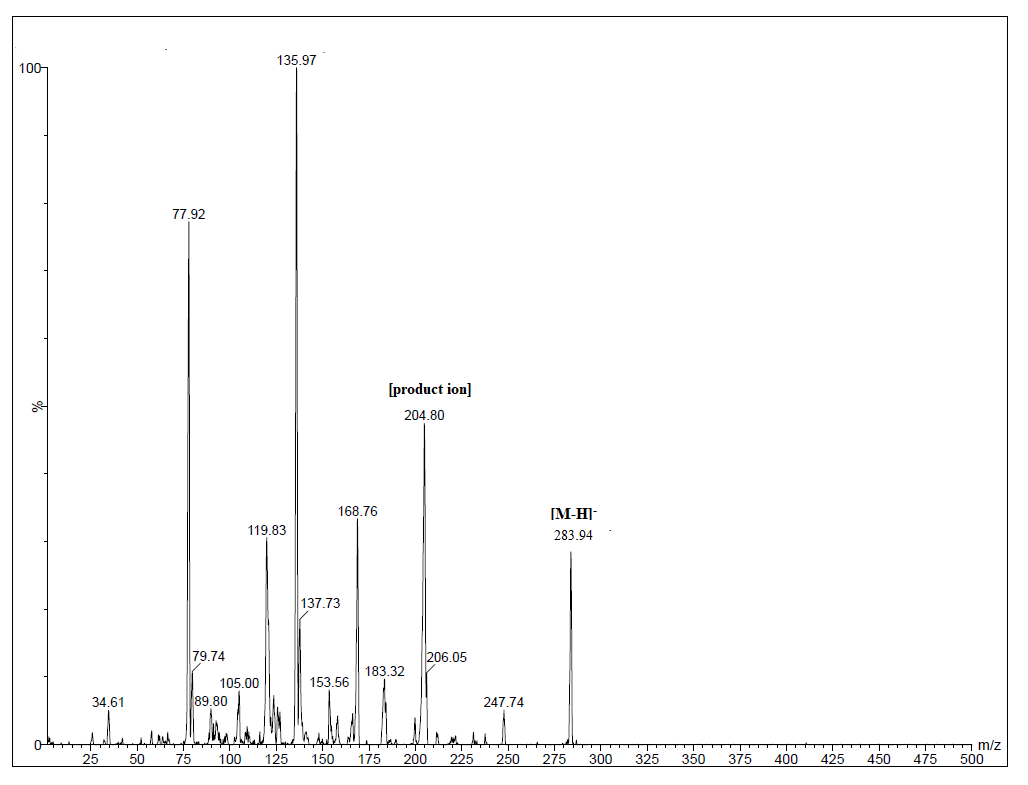


**(b)**

**Figure S1.** Representative mass spectra at negative mode ([M-H]^‑^) of (a) CT and (b) DSA.


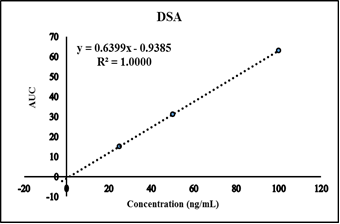

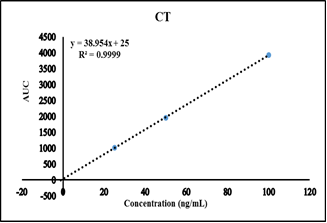

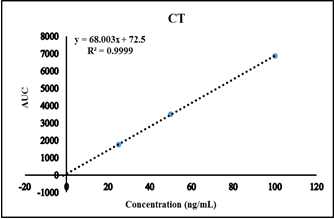

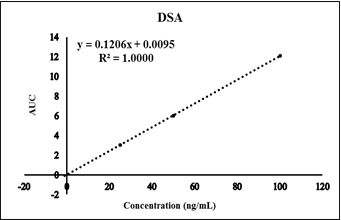


**(b)**

**(a)**


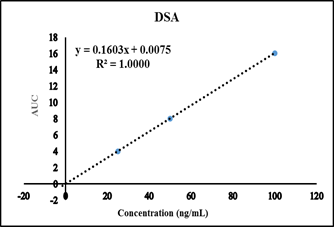

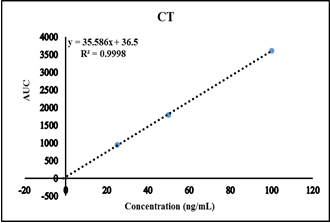


**(c)**

**Fig. S2.** Standard addition plots for determination of (a) CT and (b) DSA, in (a) Exforge HCT^®^ Tablets, (b) Atenoretic^®^ capsules and (c) Atacand Plus^®^ Tablets.


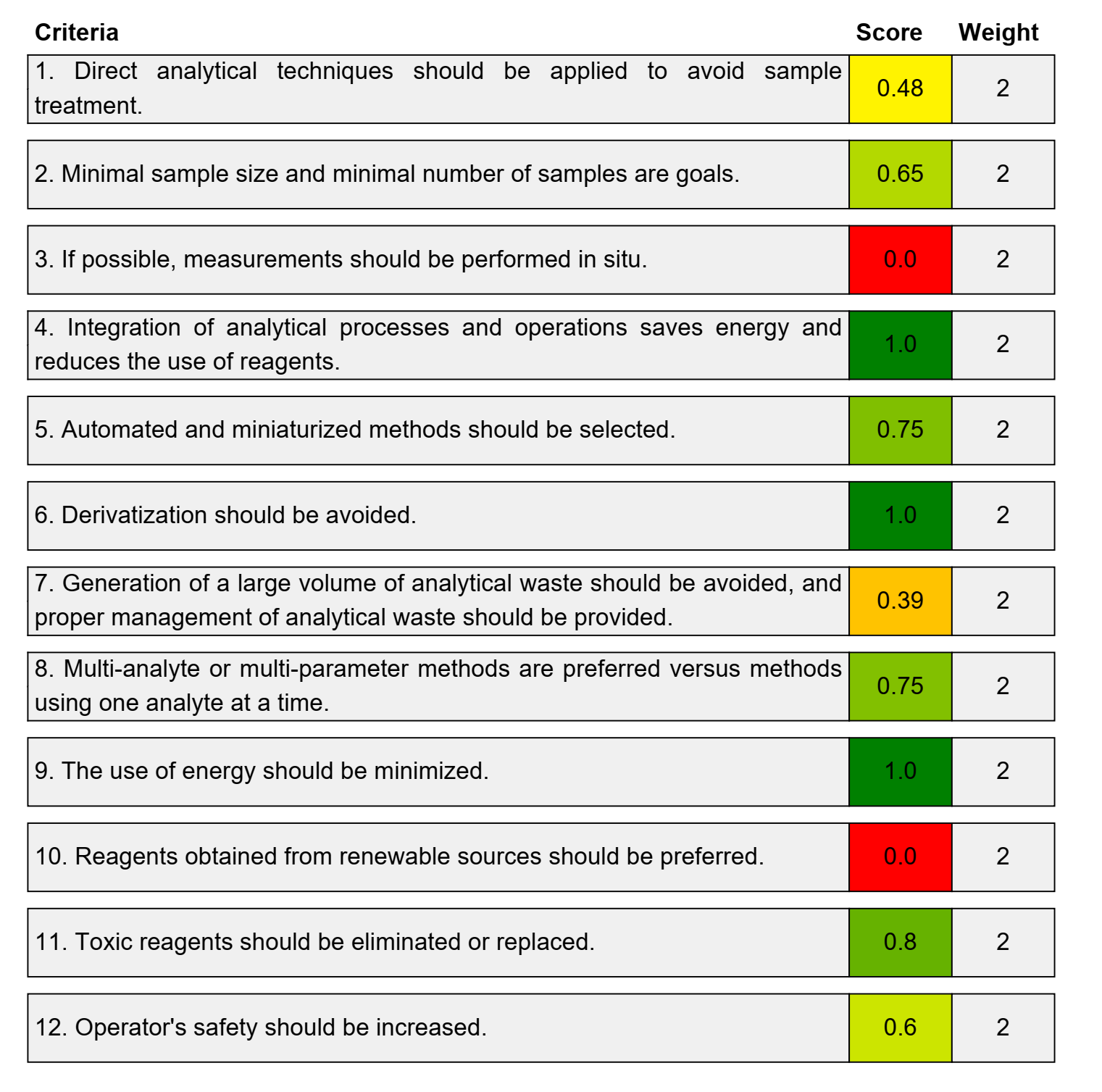


**Figure S3.** AGREE report with detailed scores for HPLC-DAD method obtained from AGREE software.


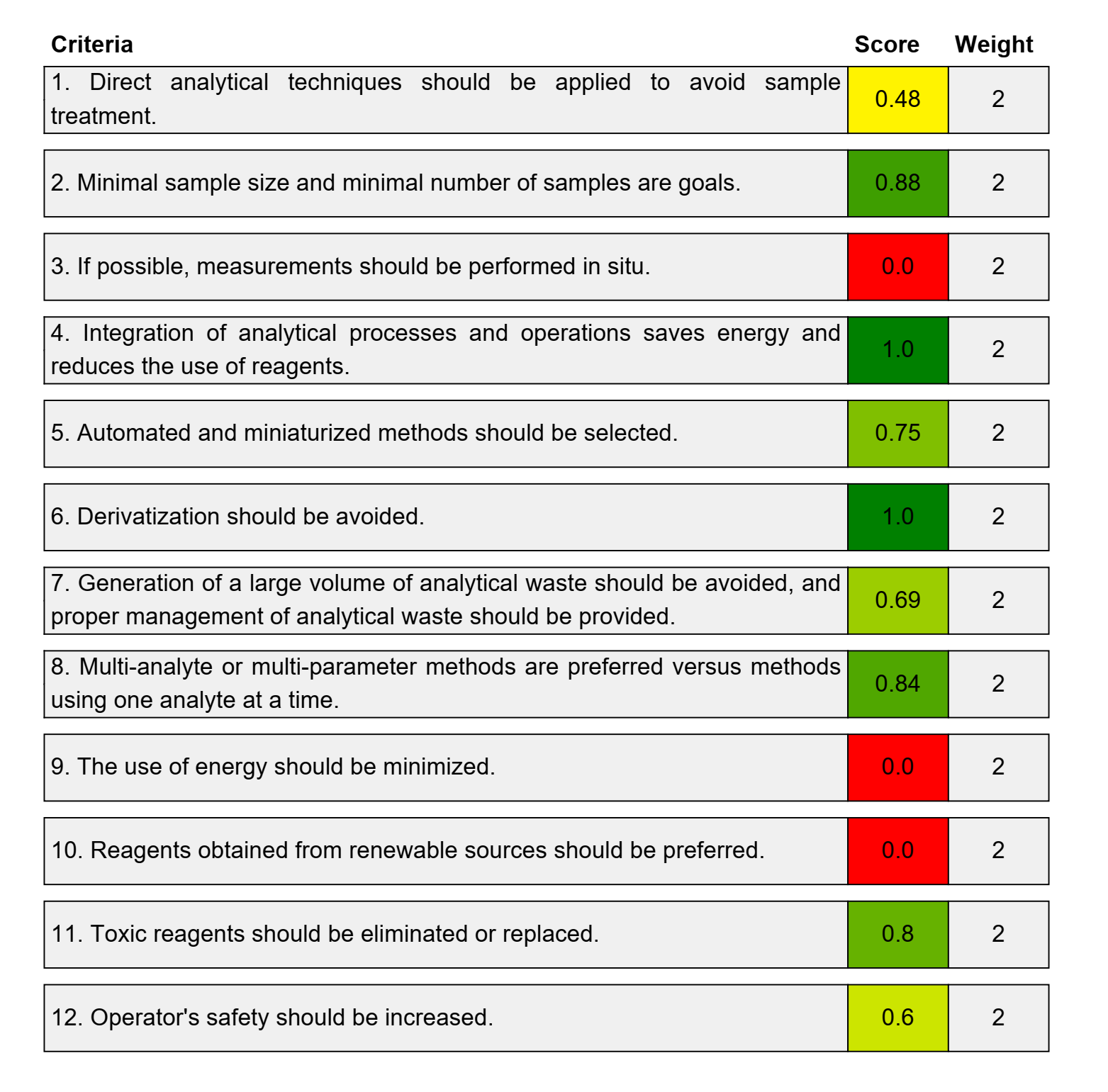


**Figure S4.** AGREE report with detailed scores for LC-MS/MS method obtained from AGREE software


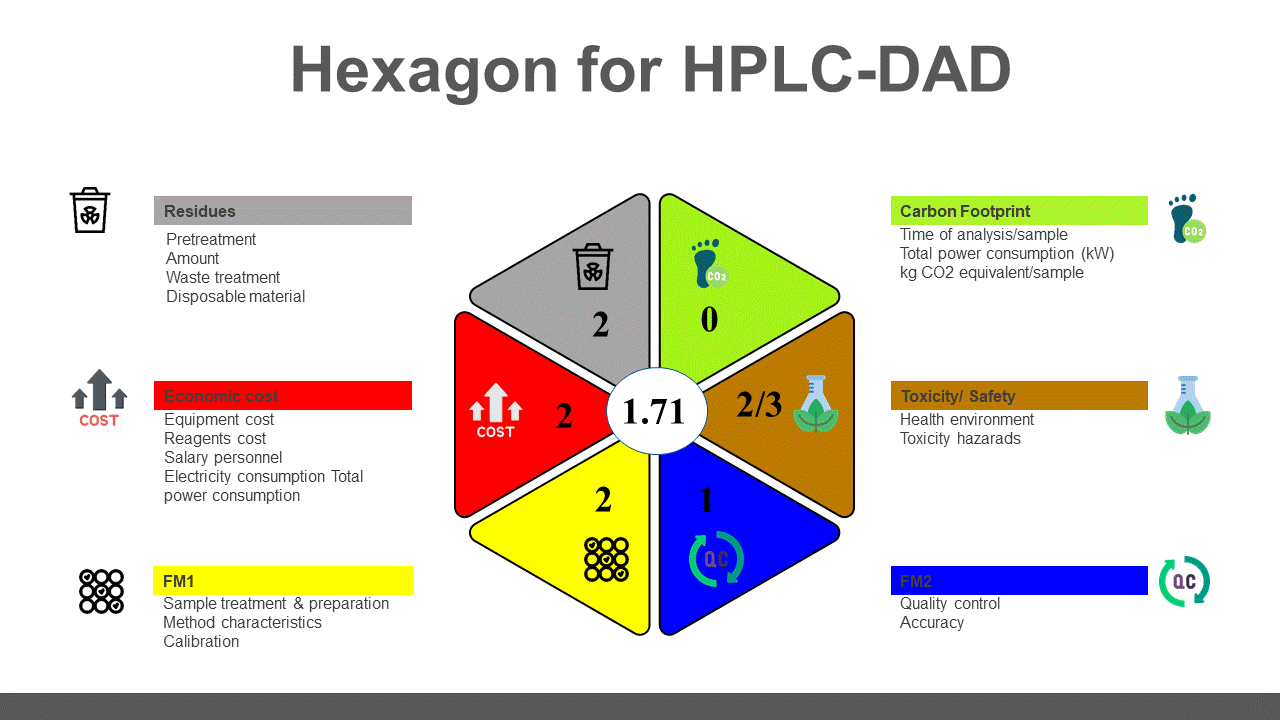

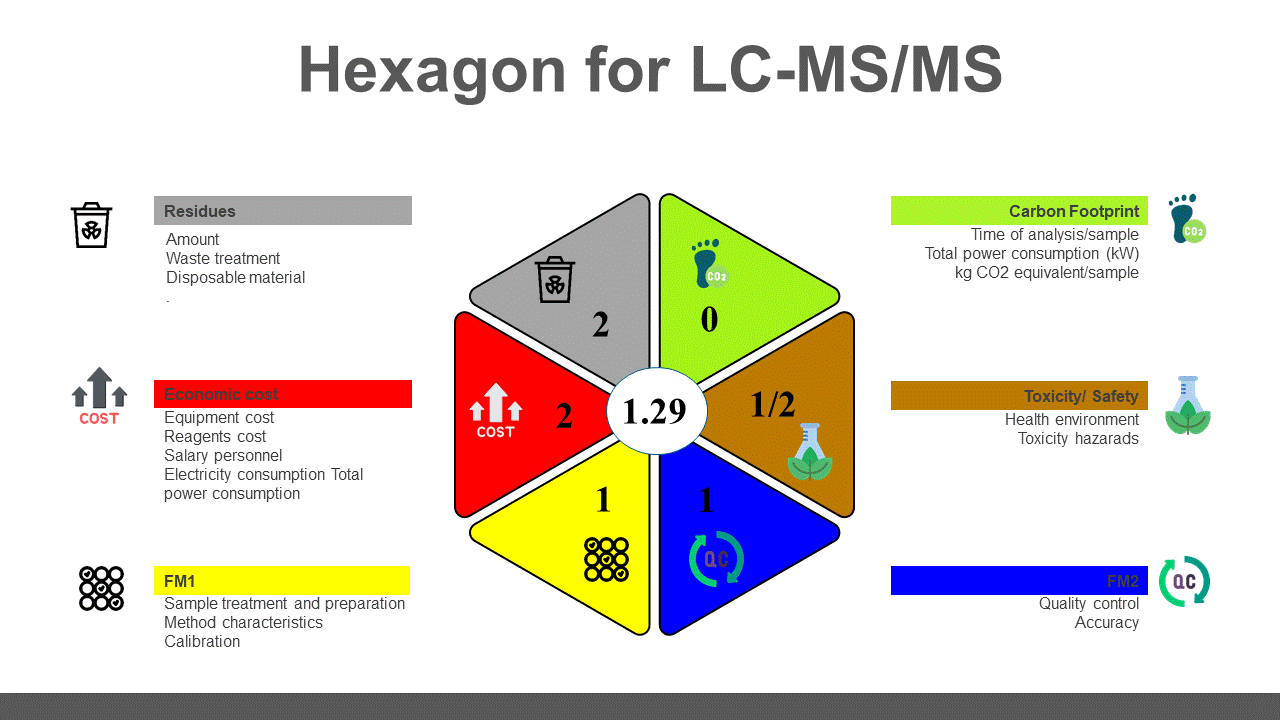
**Fig S5**. Hexagon results for HPLC-DAD and LC-MS/MS methods and the mean average in the middle circle.


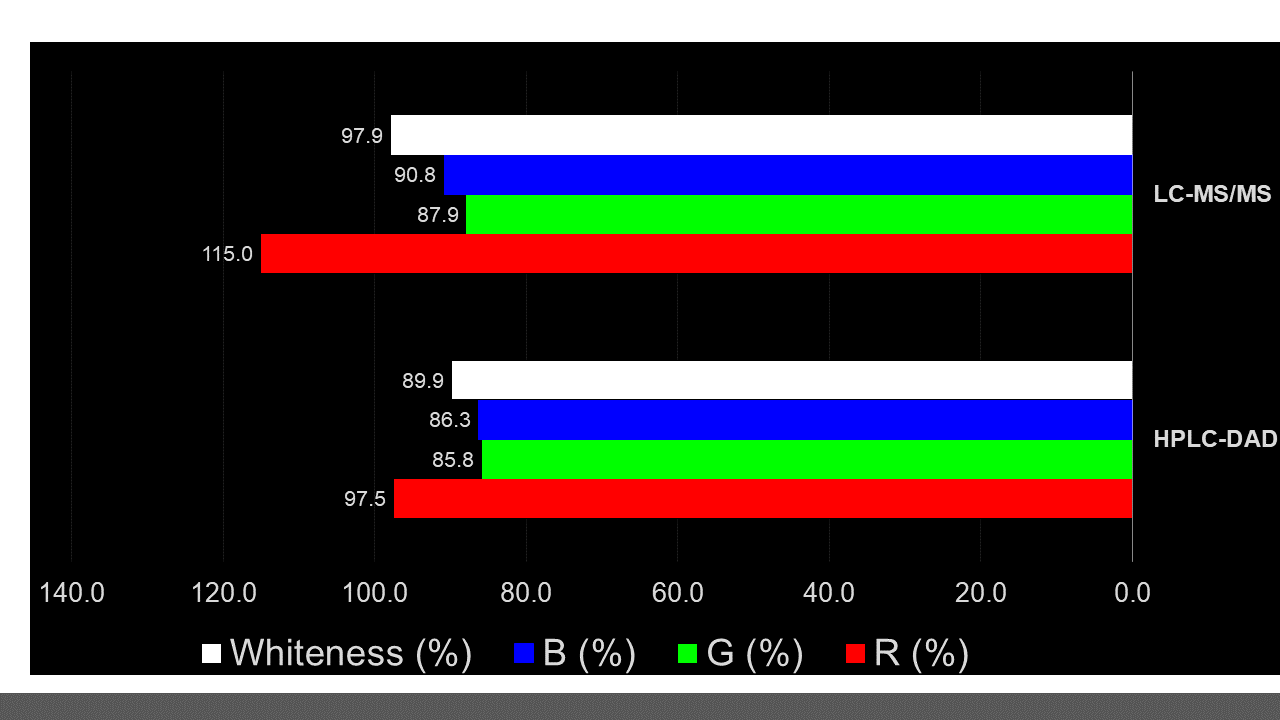


**Fig. S6**: The white line represents 100%, which means that the major assessment results from the WAC analysis are fully appropriate for the intended use. The values above 100 suggest the presence of additional capabilities above what is currently required.

**Table S1.** Determination of the proposed drugs in the laboratory-prepared mixtures by the proposed HPLC-DAD method.

| **Ratio** | **Concentrations**  **(µg/mL)** | **VAL** | **AML** | **AMI** | **ATE** | **CAN** | **HCT** | **CT** | **DSA** |
| --- | --- | --- | --- | --- | --- | --- | --- | --- | --- |
| **VAL: AMLO: HCT: CT: SAL** | | | | | | | | | |
| (640:20:50: 1:1) | 32:1:2.5:0.1:0.1 | 99.12 | 98.37 | ----- | ----- | ----- | 100.17 | 98.55 | 98.78 |
| (160:10:25: 1:1) ^*^ | 16:1:2.5:0.1:0.1 | 98.37 | 99.42 | ----- | ----- | ----- | 99.02 | 99.68 | 98.91 |
| (100:50:100: 1:1) | 10:5:10:0.1:0.1 | 98.57 | 99.74 | ----- | ----- | ----- | 98.29 | 100.29 | 101.02 |
| **AMILO: ATENO: HCT: CT: SAL** | | | | | | | | | |
| (25:500:250: 1:1) * | 2.5:50:25:0.1:0.1 | ----- | ----- | 99.57 | 98.24 | ----- | 98.29 | 100.80 | 99.47 |
| (10:200:100: 1:1) | 1:20:10:0.1:0.1 | ----- | ----- | 99.78 | 98.45 | ----- | 98.36 | 99.11 | 99.54 |
| (100:700:400: 1:1) | 10:70:40:0.1:0.1 | ----- | ----- | 99.98 | 100.09 | ----- | 98.95 | 99.68 | 99.33 |
| **CAND: HCT:CT:SAL** | | | | | | | | | |
| (160:125:1:1) * | 16:12.5:0.1:0.1 | ----- | ----- | ----- | ----- | 99.48 | 98.66 | 99.68 | 100.16 |
| (200:100:1:1) | 10:5:0.1:0.1 | ----- | ----- | ----- | ----- | 100.15 | 98.87 | 98.28 | 100.32 |
| (200:200: 1:1) | 20:20:0.1:0.1 | ----- | ----- | ----- | ----- | 98.72 | 100.09 | 100.52 | 100.46 |
| Laboratory prepared mixtures ^a^ **(Mean% ± SD):** | | 98.69 ± 0.39 | 99.18 ±  0.71 | 99.77 ±  0.20 | 98.93 ± 1.01 | 99.45 ±  0.72 | 98.97 ± 0.72 | 99.62 ± 0.86 | 99.78 ± 0.75 |

^a^ Average of three laboratory prepared mixtures.

*Ratio present in D.F.

**Table S2**: Total results of the parameters for the suggested approaches, according to the ranges of penalty points for the HEXAGON evaluation tool.

| **Parameter** | **HPLC-DAD** | **LC-MS/MS** |
| --- | --- | --- |
| **Figures of merit 1**  **(Sample treatment, Method characteristics and Calibration)** | 25  (2) | 14  (1) |
| **Figures of merit 2**  **(Quality control and Accuracy)** | 14  (1) | 6  (1) |
| **Toxicity** | 41  (2) | 25  (1) |
| **Safety** | 10  (3) | 8  (2) |
| **Residues**  **(Generated waste)** | 13  (2) | 7  (2) |
| **Carbon foot print**  **(Energy Consumption)** | Environmental impact evaluation = 0.029  (0) | Environmental impact evaluation = 0.04  (0) |
| **Economic Cost**  **(in EURO)** | 15000-30000  (2) | 15000-30000  (2) |

Total penalty points for each parameter are represented by the first numbers. The final score shown in the hexagon pictogram is indicated by the number in brackets.
